# Time-Optimal Asymmetric S-Curve Trajectory Planning of Redundant Manipulators under Kinematic Constraints

**DOI:** 10.3390/s23063074

**Published:** 2023-03-13

**Authors:** Tianyu Liu, Jingkai Cui, Yanhui Li, Siyuan Gao, Mingchao Zhu, Liheng Chen

**Affiliations:** 1 CAS Key Laboratory of On-Orbit Manufacturing and Integration for Space Optics System, Changchun Institute of Optics, Fine Mechanics and Physics, Chinese Academy of Sciences, Changchun 130033, China; 2 School of Optoelectronics, University of Chinese Academy of Sciences, Beijing 100049, China

**Keywords:** asymmetric S-curve velocity scheduling, trajectory planning, redundant manipulators, whale optimization algorithm, kinematic constraints

## Abstract

This paper proposes a novel trajectory planning algorithm to design an end-effector motion profile along a specified path. An optimization model based on the whale optimization algorithm (WOA) is established for time-optimal asymmetrical S-curve velocity scheduling. Trajectories designed by end-effector limits may violate kinematic constraints due to the non-linear relationship between the operation and joint space of redundant manipulators. A constraints conversion approach is proposed to update end-effector limits. The path can be divided into segments at the minimum of the updated limitations. On each path segment, the jerk-limited S-shaped velocity profile is generated within the updated limitations. The proposed method aims to generate end-effector trajectory by kinematic constraints which are imposed on joints, resulting in efficient robot motion performance. The WOA-based asymmetrical S-curve velocity scheduling algorithm can be automatically adjusted for different path lengths and start/end velocities, allowing flexibility in finding the time-optimal solution under complex constraints. Simulations and experiments on a redundant manipulator prove the effect and superiority of the proposed method.

## 1. Introduction

Trajectory planning is a pivotal technique for motion control, determining the efficiency and quality of task execution in electromechanical systems using robots, computerized numerical control (CNC) machine tools, and automatic machines. Interpolation and velocity scheduling can be performed separately in trajectory planning when the task specifies a path. The non-uniform rational B-spline (NURBS) interpolation technique has emerged as a research trend owing to its excellent ability to shape expression and local modification [1,2,3,4], compensating for the shortage of linear and circular interpolation adopted in robots and CNC machines [5,6].

Various velocity-scheduling approaches, also called acceleration and deceleration (Acc/Dec) control algorithms, have been explored to cooperate with interpolation modules and generate feasible trajectories for electromechanical systems along specified paths [7]. From an industrial application perspective, boosting productivity increases economic benefits, making time optimality a major research goal in motion planning. A constant velocity profile with a controlled start–stop phase is recognized as the time-optimal trajectory within the velocity and acceleration limitations of the end-effector. The trapezoidal velocity profile, a linear segment (constant velocity) with parabolic blends, is a common time-optimal trajectory in industrial robots owing to its computational simplicity [8]. However, residual vibrations in mechanical systems cause a conflict between the speed and smoothness of the robot’s motion. The inherent acceleration discontinuity of the trapezoidal velocity profile brings infinite jerks, causing undesired vibrations.

The motion profile with confined jerks obtained using the S-curve Acc/Dec control algorithm has lower residual vibrations than the trapezoidal profile. The S-curve velocity profiles have been widely adopted to generate fast and smooth motion using methods such as finite impulse response (FIR) filtering [9,10,11], polynomial models [12], trigonometric functions [13], spline interpolation [14,15,16,17], and an optimization-based technique [18,19,20,21]. Because the process of S-curve velocity scheduling is intricate and challenging, it is investigated based on a few assumptions to simplify the calculation, the most common of which are summarized as follows.

**Assumption** **1.** *The minimum jerk and acceleration are numerically equal to the maximum jerk and acceleration, respectively [22]. Accordingly, the acceleration and deceleration blocks are symmetric, resulting in a traditional symmetric S-curve (TSS) velocity profile.*

**Assumption** **2.** *The duration of the maximum and minimum accelerations is zero [23]. It produces five-phase S-curves without constant acceleration or deceleration phases.*

**Assumption** **3.** *The path length is sufficient for maximum velocity and acceleration.*

**Assumption** **4.** *The initial and final velocity of the path is zero.*

Based on the simplification of Assumption 1, the symmetrical S-shaped motion profile can be derived, which is critical in the smooth trajectory planning method. FIR filters are widely used in the motion control of automation equipment owing to the ease of hardware implementation. Based on FIR filters, the acceleration continuous motion profile was generalized to a generic model with high-order continuity [24], and a time-optimal characteristic of the reference signal was injected [25]. The S-curve of a cubic polynomial can contribute to a tradeoff between motion smoothness and computational complexity. In [26], a time-optimal S-curve trajectory planning approach based on a third-order polynomial model was reported. The motion will be smoother if the snap (the first-order derivative of jerks) or high-order derivative of jerk is limited. Thus, a high-order continuous trajectory is realized by increasing the degree of the polynomial model [27] but causes the Runge phenomenon. The trigonometric function is also an alternative for generating fast movements with low vibration [28], where the jerk can be designed as a sine [29] or cosine waveform [30]. In [22], comparisons between the polynomial and trigonometric S-curve models were presented, and a modified sigmoid function was proposed as jerks to derive the infinite-order continuous motion profile.

**Figure 1:**
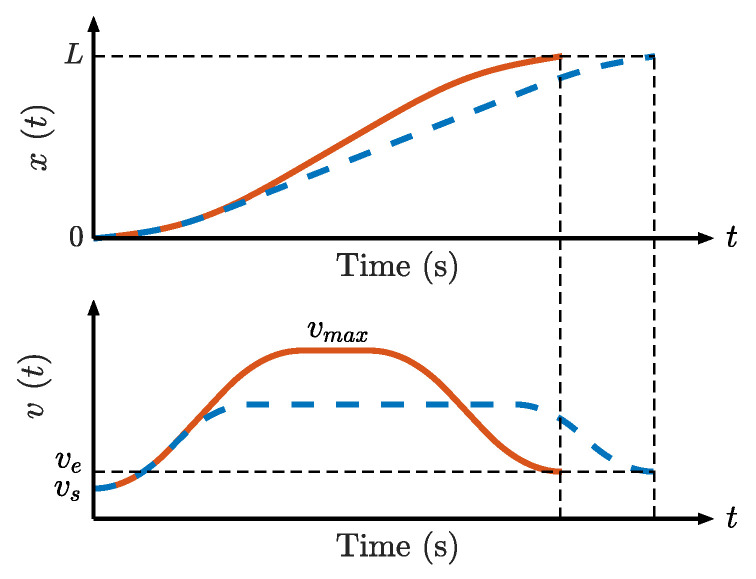
Comparison between the seven-phase and five-phase S-curves. Solid red line: seven-phase S-curve; dashed blue line: five-phase S-curve. vs. and *v_e_* are the velocity at the start and end points, respectively. *v*_max_ is the maximum velocity.

Researchers have recently focused on asymmetric S-curves to reduce overshoot and suppress residual vibrations. For the seven-phase symmetrical S-curve, a jerk ratio is added to the deceleration block, differentiating it from the acceleration block; however, the jerk of each block remains equal [31]. Without Assumption 1, the derivation of the AS motion profile that empowers different jerks in each phase becomes more complicated. Some researchers have adopted Assumption 2 for simplification, resulting in a five-phase S-shaped motion profile. The velocity scheduling process is a constrained optimization problem handled by a non-linear programming solver to minimize the execution time [32]. The metaheuristic algorithm can also be applied because of its high computational efficiency and fast convergence. The time-optimal Acc/Dec method for multiple segments with the particle swarm optimization algorithm was introduced in [23] and applied to five-axis machining in [33]. As shown in Figure 1, though the five-phase S-curve is easier to derive formulas than the seven-phase profile, it expends more time when a constant acceleration phase is required to reach the maximum velocity. In [34], based on Assumptions 3 and 4, the rest-to-rest seven-phase freeform S-curve velocity planning method was developed to balance execution efficiency and accuracy for a single-segment path.

In addition, unlike CNC machines with simple kinematics, the relationship between the end effector and joint actuators in serial manipulators is non-linear. The smooth motion profile of the end-effector may lead to infeasible joint trajectories, particularly for redundant manipulators with infinite sets of inverse kinematics solutions. A bidirectional look-ahead (BLA) trajectory planning approach was advocated in [35] to reduce the end-effector velocity based on the velocity and acceleration constraints of the joint actuators but with increased execution time. BLA is a conservative strategy. It concentrates on the dangerous paths where constraint violations occur. However, BLA neglects to explore the potential of robots when joint actuators are far from the kinematic constraints. There is an extended execution time due to the conservative joint movements.

This study proposes a kinematic constraint conversion method from joint to operation space, reshaping the end-effector velocity-limit curve along the specified path. The optimization-based asymmetric S-curve velocity scheduling is performed on each path segment which is divided at the minimum of the velocity-limit curve. The WOA proposed in [36] is adopted to find the optimal traveling time of the motion profile. The advantages of the proposed method are summarized as follows.

- The end-effector trajectory is essentially designed by the kinematic constraints of joint actuators rather than the operational-space constraints, which are used for the velocity scheduling of CNC machines [37].
- This study provides an exact velocity-limit curve of the end-effector, not conservative as BLA described in [35]. It helps to explore the maximum capabilities within the kinematic constraints of robots, generating time-optimal trajectories to improve productivity.
- The meta-heuristic WOA is adopted to find the time-optimal solution. The optimization-based trajectory planning method can flexibly handle complex constraints of the redundant manipulator and avoid solving the deceleration point.
- The jerk-limited S-shaped velocity profile is categorized into four types depending on the existence of acceleration of deceleration blocks. The WOA-based asymmetric S-curve velocity Acc/Dec can be automatically adjusted for different arc lengths and the starting and ending velocities of a path.

The generated end-effector trajectory is smooth and time-optimal, enabling the maximum capabilities of robots to be unleashed while satisfying both end-effector limits and joint kinematic constraints.

The remainder of this paper is organized as follows. Section 2 proposes the seven-phase asymmetric S-curve Acc/Dec algorithm with the optimization objective of minimizing time. The NURBS trajectory model and time-optimal S-curve trajectory generation method under constraints kinematic are presented in Section 3. The simulation and experimental results in Section 4 and Section 5, respectively, on the self-developed redundant manipulator demonstrate the performance of the proposed trajectory planning approach. Finally, Section 6 concludes the paper.

## 2. Optimization of the Seven-Phase Asymmetrical S-Curve Acc/Dec Algorithm

This section introduces the minimum-time velocity scheduling method under the end-effector limit. The possible S-shaped velocity distribution of the end-effector can be classified into four categories by the path information given in the operation space. For each case, an optimization model with respect to the time parameter is derived which involves formulas of the seven-phase asymmetrical S-curve Acc/Dec algorithm. The meta-heuristic WOA was adopted to find the optimal solution.

### 2.1. Basis of the Seven-Phase Asymmetrical S-Curve

**Figure 2:**
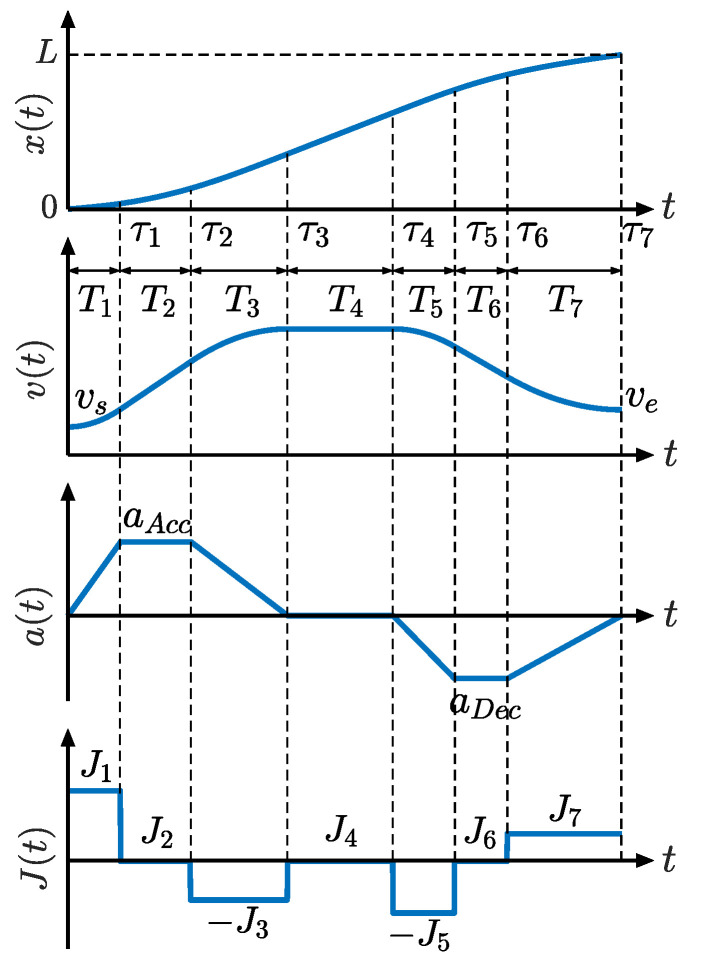
Motion profile of seven-phase asymmetric S-curve Acc/Dec.

The motion profile of the complete asymmetrical Acc/Dec, comprising seven phases, is shown in Figure 2. Phases 1–3 belong to the acceleration block, phase 4 is a constant velocity block, and phases 5–7 belong to the deceleration block.

*T_j_* (*j* = 1, 2, …, 7) represents the execution time of the *j*th phase and $\tau_{\varphi }=\sum_{j=1}^{\varphi }T_{j}\;\left(\varphi =1,2,\;\ldots ,\;7\right)$. *J_j_* (*j* = 1, 2, …, 7) denotes the jerk magnitude of the *j*th phase. *L* is the path length. vs. and *v_e_* are the velocity at the start and end points, respectively. *a_Acc_* and *a_Dec_* are the maximum accelerations in the acceleration and deceleration blocks, respectively:

(1) $$a_{Acc}=J_{1}T_{1}=J_{3}T_{3}$$

(2) $$a_{Dec}=J_{5}T_{5}=J_{7}T_{7}$$

According to the acceleration-time curve in Figure 2, *a*(*t*) can be formulated as follows:

(3) $$a\left(t\right)=\left\{\begin{array}{lll} J_{1}t & , & 0\leq t\leq \tau_{1}\\ J_{1}\tau_{1} & , & \tau_{1}\leq t\leq \tau_{2}\\ -J_{3}t+J_{3}\tau_{2}+J_{1}\tau_{1} & , & \tau_{2}\leq t\leq \tau_{3}\\ 0 & , & \tau_{3}\leq t\leq \tau_{4}\\ -J_{5}t+J_{5}\tau_{4} & , & \tau_{4}\leq t\leq \tau_{5}\\ -J_{5}\tau_{5}+J_{5}\tau_{4} & , & \tau_{5}\leq t\leq \tau_{6}\\ J_{7}t-J_{7}\tau_{6}-J_{5}\tau_{5}+J_{5}\tau_{4} & , & \tau_{6}\leq t\leq \tau_{7} \end{array}\right.,$$

The velocity–time relationship *v*(*t*) can be derived from its integral relationship with acceleration. The equations for *v*(*t*), written as design parameters, are given in Equation (A1) in Appendix A. The integration of the velocity yields the displacement-time relationship *x*(*t*), and the corresponding equations are derived in Equation (A2) in Appendix A.

The total displacement ${x(\tau }_{7})$ should be equal to the path length *L*. The velocity at the last instant should be equal to the ending velocity. Thus, the following constraints at the last point are formed:

(4) $$v\left(\tau_{7}\right)=v_{e},x\left(\tau_{7}\right)=L.$$

Moreover, the velocity and acceleration at an arbitrary instant should be less than the end-effector motion limits, which gives the constraints of the design parameters as follows:

(5) $$a\left(t\right)\leq a_{\max},$$

(6) $$v\left(t\right)\leq v_{\max}.$$

where *a*_max_ and *v*_max_ are the maximum acceleration and velocity of the end effector, respectively.

### 2.2. Parameters Design of the Seven-Phase Asymmetrical S-Curve Acc/Dec

If the given path length *L* is sufficiently long, an S-curve motion profile with seven full phases can be obtained. However, one or more of these seven phases probably has zero duration. The S-curve may not simultaneously have acceleration, constant velocity, or deceleration blocks if the distance between the start and end points is short. Thus, the first step is to check the travel distance by defining the reference length $L_{\mathrm{ref}}{\;=L}_{\mathrm{Acc}}{+L}_{\mathrm{Dec}}$, where:

(7) $$L_{Acc}=\left\{\begin{array}{lll} \left(v_{s}+v_{\max}\right)\sqrt{\frac{v_{\max}-v_{s}}{J_{\max}}} & , & v_{\max}-v_{s}\leq \frac{A_{\max}^{2}}{J_{\max}}\\ \frac{1}{2}\left(v_{s}+v_{\max}\right)\left(\frac{v_{\max}-v_{s}}{J_{\max}}+\frac{A_{\max}}{J_{\max}}\right) & , & v_{\max}-v_{s}>\frac{A_{\max}^{2}}{J_{\max}} \end{array}\right.$$

(8) $$L_{Dec}=\left\{\begin{array}{ccc} \left(v_{\max}+v_{e}\right)\sqrt{\frac{v_{\max}-v_{e}}{J_{\max}}} & , & v_{\max}-v_{e}\leq \frac{A_{\max}^{2}}{J_{\max}}\\ \frac{1}{2}\left(v_{\max}+v_{e}\right)\left(\frac{v_{\max}-v_{e}}{J_{\max}}+\frac{A_{\max}}{J_{\max}}\right) & , & v_{\max}-v_{e}>\frac{A_{\max}^{2}}{J_{\max}} \end{array}\right.$$

in which, *J*_max_, *A*_max_, and *v*_max_ are the end-effector jerk, acceleration, and velocity limits, respectively. $L_{\mathrm{Acc}}$ and $L_{\mathrm{Dec}}$ represent the reference length of the acceleration and deceleration block, respectively, which are calculated by trapezoidal approximation to the corresponding area of the velocity-time curve in Figure 2. For more details, please refer to [37].

Type 1. If $L\geq L_{\mathrm{ref}}$, a long distance is identified. Setting ${v(\tau }_{7}{)=\;v}_{e},{\;x(\tau }_{7})\;=L$ based on Equation (4), the jerk of each phase can be derived as follows:

(9) $$\left\{\begin{array}{l} J_{1}=\frac{2}{T_{1}}\cdot \frac{3C_{T}L-(D_{T}+3C_{T}T_{7})v_{e}-(E_{T}+3C_{T}\tau_{5})v_{s}}{C_{T}(B_{T}+3A_{T}(\tau_{5}-T_{1}))+A_{T}E_{T}}\\ J_{5}=\frac{2}{T_{5}}\cdot \frac{3A_{T}L-(B_{T}+3A_{T}(\tau_{7}-T_{1}))v_{e}+(B_{T}-3AT_{1})v_{s}}{C_{T}(B_{T}+3A_{T}(\tau_{5}-T_{1}))+A_{T}E_{T}}\\ J_{3}=J_{1}T_{1}/T_{3}\\ J_{7}=J_{5}T_{5}/T_{7}\\ J_{2}=J_{4}=J_{6}=0 \end{array}\right.,$$

where:

(10) $$\left\{\begin{array}{l} A_{T}=T_{1}+2T_{2}+T_{3}\\ B_{T}=T_{1}^{2}-3T_{2}^{2}-T_{3}^{2}-3T_{2}T_{3}\\ C_{T}=T_{5}+2T_{6}+T_{7}\\ D_{T}=T_{5}^{2}+3T_{6}^{2}-T_{7}^{2}+3T_{5}T_{6}\\ E_{T}=-T_{5}^{2}+3T_{6}^{2}+T_{7}^{2}+3T_{6}T_{7} \end{array}\right..$$

According to the jerk-time curve in Figure 2, *J*_2_, *J*_4_, and *J*_6_ are always zero. From Equations (1) and (2), *J*_3_ and *J*_7_ are related to *J*_1_ and *J*_5_, respectively. Thus, there are two unknowns, *J*_1_ and *J*_5_, which can be uniquely determined by Equation (4) of velocity and path length constraints.

Type 2. If ${L\;<\;L}_{\mathrm{ref}}$, the travel length is recognized as short. We further determine if an acceleration or deceleration block exists through the relationship between the starting and ending velocities as follows. Type 2.1 does not contain a deceleration block, so there is an unknown *J*_1_. If acceleration is absent, the only unknown is *J*_5_, which corresponds to Type 2.2. The constraints (4) are impossible to satisfy simultaneously. The path length should be guaranteed primarily. Thus, a relaxed velocity constraint is proposed in that the calculated velocity should be less than the given one at the ending point. Moreover, neither acceleration nor deceleration blocks exist in Type 2.3. The motion profile of the constant velocity block is exclusive once the path length and start velocity are determined.

Type 2.1. If $v_{s}{\;<\;v}_{e}$, there is no deceleration block, that is $T_{5}\;{=T}_{6}\;{=T}_{7}\;=0$. Setting $x\left(\tau_{4}\right)=\;L$ based on Equation (4), the jerk of each phase can be expressed as follows:

(11) $$\left\{\begin{array}{l} J_{1}=\frac{6}{T_{1}}\cdot \frac{L-(T_{1}+T_{2}+T_{3}+T_{4})v_{s}}{T_{1}^{2}-T_{3}^{2}+3T_{2}T_{4}+3(T_{1}+T_{2}+T_{3})(T_{2}+T_{3}+T_{4})}\\ J_{3}=J_{1}T_{1}/T_{3}\\ J_{2}=J_{4}=J_{5}=J_{6}=J_{7}=0 \end{array}\right..$$

Accordingly, the resulting velocity of the end point $v_{e,r}$ can be derived, which cannot be higher than $v_{e}$. Thus, the constraints on the design parameters are specified as follows:

(12) $$v_{e,r}=v_{s}+\frac{3A_{T}\left(L-(T_{1}+T_{2}+T_{3}+T_{4})v_{s}\right)}{T_{1}^{2}-T_{3}^{2}+6T_{2}T_{4}+3(T_{1}+T_{2}+T_{3})(T_{2}+T_{3}+T_{4})}\leq v_{e}.$$

Type 2.2. If $v_{s}{\;>\;v}_{e}$, the acceleration block is nonexistent, that is, $T_{1}\;{=T}_{2}\;{=T}_{3}\;=0$. Setting ${\left.{x(\tau }_{7})\right|}_{T_{1}{=T}_{2}{=T}_{3}=0}=L$ based on Equation (4), the jerk of each phase can be denoted as

(13) $$\left\{\begin{array}{l} J_{5}=\frac{6}{T_{5}}\cdot \frac{L-(T_{4}+T_{5}+T_{6}+T_{7})v_{s}}{T_{5}^{2}+3T_{6}^{2}+2T_{7}^{2}+3T_{5}T_{6}+3T_{5}T_{7}+6T_{6}T_{7}}\\ J_{7}=J_{5}T_{5}/T_{7}\\ J_{1}=J_{2}=J_{3}=J_{4}=J_{6}=0 \end{array}\right..$$

The resulting end velocity should be less than $v_{e}$. The corresponding constraint imposed on the design parameters is given as:

(14) $$v_{e,r}=v_{s}-\frac{3C_{T}\left(L-(T_{4}+T_{5}+T_{6}+T_{7})v_{s}\right)}{T_{5}^{2}+3T_{6}^{2}+2T_{7}^{2}+3T_{5}T_{6}+3T_{5}T_{7}+6T_{6}T_{7}}\leq v_{e}.$$

Type 2.3. If $v_{s}{\;=v}_{e}$, the motion profile contains only the constant velocity block and all jerks are zero. In addition, the execution time of the fourth phase is expressed as $T_{4}=L/v_{s}$ based on Equation (4), and for other phases, the execution times are zero.

The jerk obtained at each phase of the S-curve should not be less than zero in all cases. The direction is reflected in the signs in Equations (15) and (16), which ensures nonzero jerks.

(15) $$J_{1}\geq 0,$$

(16) $$J_{5}\geq 0.$$

### 2.3. Principle of the WOA Algorithm

The WOA is a swarm-based meta-heuristic algorithm [36] that is competitive in industrial applications, especially for engineering optimization problems [38]. It mimics the hunting behavior of humpback whales, comprising encircling, attacking, and searching for prey. The mathematical model is described below, and a detailed description can be found in [36].

1. Encircling prey.

The WOA assumes that the current best-search agent is the target prey. The position of other search agents is updated towards the best search agent just as humpback whales encircle their prey and it is formulated as:

(17) $$\vec{D}=\left|\vec{C}\vec{X^{*}}(\delta )-\vec{X}(\delta )\right|,$$

(18) $$\vec{X}(\delta +1)=\vec{X^{*}}(\delta )-\vec{A}\cdot \vec{D},$$

where · denotes element-wise multiplication and   denotes the absolute value. *δ* denotes the current iteration, $\vec{X}$ is the position vector, and $\vec{X^{*}}$ is the position vector of the best-search agent. $\vec{A}$ and $\vec{C}$ are the coefficient vectors, defined as:

(19) $$\vec{A}=2\vec{a}\cdot \vec{r}-\vec{a},$$

(20) $$\vec{C}=2\cdot \vec{r},$$

where $\vec{a}$ decreases linearly from 2 to 0 during the iterations and $\vec{r}$ is a random vector defined in [0,1].

2. Bubble-net attack approach (exploitation).

The WOA simulates the attack behavior using two options with equal probabilities: a shrinking encircling mechanism and a spiral updating position. The related mathematical model is as follows:

(21) $$\vec{X}(\delta +1)=\left\{\begin{array}{lll} \vec{X^{*}}(\delta )-\vec{A}\cdot \vec{D} & , & p_{r}<0.5\\ \vec{D^{\prime }}\cdot e^{bl}\cdot \cos(2\pi l)+\vec{X^{*}}(\delta ) & , & p_{r}>0.5 \end{array}\right.,$$

where $p_{r}$ is a random number [0,1]. If $p_{r}\;<\;0.5$, the search agent performs the shrinking encircling mechanism. The enclosed circle gradually shrinks as the value of $\vec{a}$ decreases. Otherwise, the spiral model updates the position. $\vec{D^{\prime }}=\left|\vec{X^{*}}\left(\delta \right)-\vec{X}\left(\delta \right)\right|$ represents the distance from an arbitrary search agent to the best one, *b* is a constant that determines the shape of the logarithmic spiral, *l* is a random number in [0,1], and · denotes the element-wise multiplication.

The new position of the search agent was located between its original and prey positions when $\left|\vec{A}\right|\;<\;1$. The restricted but promising search space contributes to the exploitation capability of the WOA.

3. Search for prey (exploration).

Inspired by the random prey-seeking behavior of humpback whales, the WOA can accomplish global search by setting $\left|\vec{A}\right|>1$. The search agent is forced away from a randomly selected reference agent, which differs from the exploitation period having the best agent as the reference. The search behavior is modeled as:

(22) $$\vec{D}=\left|\vec{C}\cdot \vec{X_{rand}}-\vec{X}\right|,$$

(23) $$\vec{X}\left(\delta +1\right)=\vec{X_{rand}}-\vec{A}\cdot \vec{D},$$

where $\vec{X_{rand}}$ represents the position vector of a random search agent.

### 2.4. WOA-Based Asymmetrical S-Curve Acc/Dec

WOA is adopted to find the best duration (*T_j_*, *j* = 1,2, …, 7) for each phase of the S-curve and to minimize the execution time of a single NURBS segment. The related pseudocode is presented in Algorithm 1 and is described in detail below.
**Algorithm 1.** Pseudo-code of WOA-based asymmetrical S-curve Acc/Dec**Input:** (1) Arc length *L*, start velocity *v_s_*, and end velocity *v_e_*; (2) S-curve type. **Output:** The best search agent X*.1:Set the swarm size *N_s_*, maximum iterations *N_iter_*, and the weight factor ϑ2:Initialize the population *X_r_* of whales based on the S-curve type3:Evaluate the fitness of each search agent using Equation (25)4:Set X* as the best search agent5:**while** (*δ* < *N_iter_*)6: **for** each search agent7: Update *a*, *A*, *C*, *l*, and *p_r_*8:  **if** (*p_r_* < 0.5)9:  **if** (|*A*| < 1)10:   Calculate a new position of the current search agent using the first formula in Equation (21)11:  **otherwise** (|*A*| ≥ 1)12:   Select a random search agent (*X_rand_*)13:   Calculate a new position of the current search agent using Equation (23)14:  **end if**15:  **otherwise** (*p_r_* > 0.5)16:  Calculate a new position of the current search agent using the second formula in Equation (21)17:  **end if**18: Check if the new position respects the kinematic constraints 19: If not, discard it. Otherwise, update the current search agent to the new position20: **end for**21: Calculate the fitness of each search agent using Equation (25)22: Update X* if there is a better solution23: *δ* = *δ* + 124:**end while**25:**return **X*

1. Initialize the population of whales based on the S-curve type.

(24) $$X=\left\{X_{1},X_{2},...,X_{N_{s}}\right\},$$

where *X_r_* = {*T*_1_, *T*_2_, *T*_3_, *T*_4_, *T*_5_, *T*_6_, *T*_7_} (*r* = 1,2, …, *N_s_*) represents a potential solution, *N_s_* is the swarm size, and *T_j_*(*j* = 1,2, …, 7) is the duration of the *j*th phase of the S-shaped motion profile. If the path is recognized as a short distance, determine whether the S-curve velocity profile contains the acceleration and deceleration blocks by comparing the velocities at the start and end points and set the corresponding duration *T_j_*(*j* = 1,2, …, 7) as zero.

2. Evaluate the fitness value of each search agent.

The optimization problem is formulated as a maximal problem. For minimizing the motion time, the fitness function can be specified as:

(25) $$fitness=\frac{1}{\sum_{j=1}^{7}\vartheta_{j}T_{j}},$$

where $\vartheta_{j}$ is a weight factor imposed on the *j*th duration *T_j_*(*j* = 1,2, …, 7) of the S-curve. The best search agent, X*, is determined by comparing the fitness values. A higher value indicates a better search agent.

3. Determine whether the constraints are satisfied.

Check that the new position of search agent *X_r_* respects the maximum acceleration limits (5), maximum velocity limits (6), and jerk constraints (15) and (16). It is also necessary to judge whether the ending velocity constraint (12) or (14) is met if the S-curve is Type 2.2 or Type 2.3. The path and velocity constraints (4) do not need to be checked again since they are already considered when the designing parameters. The agent is updated to the new position if all constraints are satisfied; otherwise, no update is performed.

The WOA-based asymmetric S-curve Acc/Dec can automatically recognize the absence of acceleration, constant velocity, and deceleration blocks, depending on the path requirements. The duration of each phase of the S-curve can be different and even zero if necessary. The proposed algorithm is applicable to any cases with arbitrary path length and start–stop velocity because it depends on no assumption.

In addition, the optimization methods used to determine the optimal time for each S-curve phase are not specific to the WOA. Other global search algorithms, such as the particle swarm optimization algorithm, grey wolf optimizer, and genetic algorithm, were also suitable. The WOA offers fast computation, good convergence, flexibility, and robustness. Moreover, it has been tested in numerous engineering problems [38]; therefore, it was adopted in this study.

## 3. Time-Optimal Asymmetric S-Curve Trajectory for Redundant Manipulators

### 3.1. Trajectory Model Based on the NURBS Interpolation Technique

The NURBS path *C*(*u*) can be expressed as:

(26) $$C\left(u\right)=\frac{\sum_{i=0}^{m}\omega_{i}d_{i}N_{i,p}\left(u\right)}{\sum_{i=0}^{m}\omega_{i}N_{i,p}\left(u\right)},u\in \left[0,1\right],$$

where *p* denotes the degree of the NURBS curve; *d_i_* (*i* = 0,1, …, *m*) are the control points, a total of *m* + 1; $\omega_{i}$ is the weights corresponding to the control points that determine the influence on the parametric curve; and $N_{i,p}\left(u\right)$ is the B-spline basis functions on a non-uniform knot vector ${U=[u}_{0}{,u}_{1}{,\ldots ,u}_{m+p+1}]$. The *i*th basis function is recursively expressed as follows:

(27) $$\left\{\begin{array}{l} N_{i,0}\left(u\right)=\left\{\begin{array}{l} \begin{array}{ll} 1 & u_{i}\leq u\leq u_{i+1} \end{array}\\ \begin{array}{ll} 0 & otherwise \end{array} \end{array}\right.\\ N_{i,p}\left(u\right)=\frac{u-u_{i}}{u_{i+p}-u_{i}}N_{i,p-1}\left(u\right)+\frac{u_{i+p+1}-u}{u_{i+p+1}-u_{i+1}}N_{i+1,p-1}\left(u\right) \end{array}\right.,$$

It is stipulated that 0/0 = 0.

The interpolation parameter *u* can be discretized as *u_k_* = *u*(*kT_s_*) and calculated iteratively as follows:

(28) $$u_{k+1}=u_{k}+\frac{v_{c,k}T}{{\left|\frac{dC(u)}{du}\right|}_{u_{k}}},$$

where *T* denotes the time interval, which is initially set as the sampling time *T_s_*, and *v_c,k_* is the trial motion profile of the end-effector at the *k*th time instant.

The arc length of an interval [*u_a_*, *u_b_*] on curve *C*(*u*) is defined as:

(29) $$L\left(u\right)=\int_{u_{a}}^{u_{b}}\left|C^{\prime }\left(u\right)\right|du,$$

where *C’*(*u*) = *dC*(*u*)/*du* is the first-order derivative of Equation (26).

The solution of Equation (29) can be calculated using the composite Simpson formula if the interval is sufficiently small as follows:

(30) $$L\left(u_{a},u_{b}\right)=L\left(u_{a},\frac{u_{a}+u_{b}}{2}\right)+L\left(\frac{u_{a}+u_{b}}{2},u_{b}\right),$$

where:

(31) $$L\left(u_{a},\frac{u_{a}+u_{b}}{2}\right)=\frac{u_{b}-u_{a}}{12}\left[L^{\prime }\left(u_{a}\right)+4L^{\prime }\left(\frac{3u_{a}+u_{b}}{2}\right)+L^{\prime }\left(\frac{u_{a}+u_{b}}{2}\right)\right],$$

(32) $$L\left(\frac{u_{a}+u_{b}}{2},u_{b}\right)=\frac{u_{b}-u_{a}}{12}\left[L^{\prime }\left(\frac{u_{a}+u_{b}}{2}\right)+4L^{\prime }\left(\frac{u_{a}+3u_{b}}{2}\right)+L^{\prime }\left(u_{b}\right)\right],$$

### 3.2. Kinematic Constraint Handling

In general, the kinematic constraints of a redundant manipulator refer to the joint position, velocity, and acceleration limits, prescribed as follows:

(33) $$\begin{array}{l} q_{min,i}\leq q_{i}\leq q_{max,i}\\ {\dot{q}}_{min,i}\leq {\dot{q}}_{i}\leq {\dot{q}}_{max,i}\\ {\ddot{q}}_{min,i}\leq {\ddot{q}}_{i}\leq {\ddot{q}}_{max,i} \end{array},$$

where $q_{\max,i}{\;(q}_{\min,i}{),\dot{q}}_{\max,i}{\;(\dot{q}}_{\min,i}),\;\mathrm{and}\;{\ddot{q}}_{\max,i}\;({\ddot{q}}_{\min,i})$ are the maximum (minimum) joint position, velocity, and acceleration of the *i*th joint, respectively; *i* = 1, …, *n*, *n* is the number of joints.

As is well known that the kinematic mapping of a manipulator is non-linear between the configuration and operation spaces. The S-shaped motion profile which schedules in operation space based on end-effector limits, that is, the end-effector trial motion profile, may lead to constraint violations in the configuration space. Assuming all joints are successfully confined within the maximum feasible position using the redundancy property, joint actuators may still violate the velocity and acceleration constraints. Thus, the S-curve trajectory designed by end-effector limits yields a set of trial joint trajectories that may be infeasible.

Although the trial joint trajectories cannot be directly used as input to control joint servos, it can be treated as a priori to derive the trial velocity and acceleration values of the *i*th joint as follows:

(34) $${\dot{q}}_{k+1,i}=\frac{q_{k+1,i}-q_{k,i}}{T},$$

(35) $${\ddot{q}}_{k+1,i}=\frac{q_{k+1,i}-2q_{k,i}+q_{k-1,i}}{T^{2}}.$$

According to the last two inequation constraints in Equation (33), the scaling time interval, indicating the minimum allowed time to move from $q_{k}$ to $q_{k+1}$ when satisfying all joint velocity and acceleration constraints, can be derived by solving the following optimization problem:

(36) $$\begin{array}{ll} \underset{1/T_{k}}{\min}-\frac{1}{T_{k}} & \\ \mathrm{subject}\;\mathrm{to}\;T_{k}>0 & \\ & {\dot{q}}_{\min,i}\leq \frac{q_{k+1,i}-q_{k,i}}{T_{k}}\leq {\dot{q}}_{\max,i},\forall i=1,...,n\\ & {\ddot{q}}_{\min,i}\leq \frac{q_{k+1,i}-2q_{k,i}+q_{k-1,i}}{T_{k}^{2}}\leq {\ddot{q}}_{\max,i},\forall i=1,...,n \end{array},$$

in which *T_k_* is the *k*th scaling time interval; $q_{k}$ and $q_{k+1}$ are the joint position at the *k*th and (*k* + 1)th time instant, respectively; and *n* is the degree of freedom, which is equal to the number of joints of the redundant manipulator. The angle of joint motion from $q_{k}$ to $q_{k+1}$ is certain. If the joint velocity or acceleration exceeds the corresponding limit, the time interval *T_k_* should be increased which is determined by the joint that exceeds the limit the most. Otherwise, *T_k_* can be decreased. It means the joint actuators are allowed to improve motion capabilities before reaching the acceleration or velocity boundary.

The joint motor has the same motion capability in both directions, and the joint velocity and acceleration limits can be rewritten as ${\dot{q}}_{\min}{=-\dot{q}}_{\lim}{,\dot{q}}_{\max}{=\dot{q}}_{\lim}\;\mathrm{and}\;{\ddot{q}}_{\min}=-{\ddot{q}}_{\lim},\;{\ddot{q}}_{\max}={\ddot{q}}_{\lim}$, respectively. By setting $\Gamma_{k}=1/T_{k}$, Equation (36) can be converted into the following non-linear optimization form:

(37) $$\begin{array}{ll} \underset{\Gamma_{k}}{\min}-\Gamma_{k} & \\ \mathrm{subject}\;\mathrm{to}\;-\Gamma_{k}<0 & \\ & \left|q_{k+1}-q_{k}\right|\cdot \Gamma_{k}-{\dot{q}}_{lim}\leq 0\\ & \left|q_{k+1}-2q_{k}-q_{k-1}\right|\cdot \Gamma_{k}^{2}-{\ddot{q}}_{lim}\leq 0 \end{array},$$

An interior-point algorithm for non-linear programming [39] was adopted to solve Equation (37). Based on the calculated *k*th time interval *T_k_*, the end-effector velocity was updated as follows:

(38) $$v_{\lim,k}=v_{c,k}T_{s}/T_{k},$$

If the *k*th time interval *T_k_* is greater than the sampling time *T_s_*, implying constraint violations from $q_{k}$ to $q_{k+1}$, the end-effector velocity should be reduced to comply with the kinematic constraints of the joint actuators. In contrast, the robot has not released its full potential if *T_k_* < *T_s_*.

The velocity-limit curve (VLC) as a function of the path, which represents the maximum tolerable velocity of the end-effector under the kinematic bounds of the joint actuators, can be treated as a constraint to performing velocity scheduling. Thus, the end-effector maximum velocity constraint (6) can be rewritten as:

(39) $$v\left(t\right)\leq v_{\lim}.$$

### 3.3. Time-Optimal Asymmetric S-Curve for Multiple NURBS Segments

Based on the VLC of the end-effector, the critical points where the kinematic constraints of the joint actuators exceed the maximum can be found. The NURBS path is split at these critical points, and velocity scheduling is performed for each segment using the asymmetric S-curve Acc/Dec based on WOA. The pseudocode in Algorithm 2 summarizes this process.

The minimum execution time along the desired path minimizes the execution time for each NURBS segment. If the NURBS curve is divided into *N_γ_* segments, the arc length *L_γ_* (*γ* = 1,2, …, *N_γ_*) of each segment can be recursively calculated using Equations (29)–(32). The start and end velocities of each segment were determined using the VLC of the end-effector in Equation (38). Based on the method presented in Section 2.2, the S-curve type along the *γ*th NURBS segment can be determined. Thus, each segment can be viewed as a single path to schedule the velocity. The WOA-based asymmetric S-curve Acc/Dec algorithm generates a time-optimal velocity profile for each segment. The time-optimal asymmetric S-curve trajectory for the end-effector which respects all constraints is generated eventually.
**Algorithm 2**. Time-optimal asymmetric S-curve trajectory generation**Input:** VLC of the end-effector along the specified path. **Output:** The end-effector desired motion profile.1:Identify the critical points at local minimum points of VLC in Equation (38)2:Split the NURBS curve into *N_γ_* segments at critical points3:Estimate the arc length of each NURBS segment using Equations (29)–(32)4:Derive the start and end velocities of each segment using the VLC in Equation (38)5:**for** each NURBS segment6: Calculate the reference length using Equations (7) and (8)7: **if** *L_γ_* ≥ *L_ref,γ_*8:  Call Algorithm 19:  Calculate the jerk of each phase of the S-curve using Equation (9)10: **otherwise** *L_γ_* < *L_ref,γ_*11:  Determine whether the acceleration and deceleration blocks exist12  **if**
*v_s,γ_* < *v_e,γ_*13:   The deceleration block does not exist14:   Set *T*_5,*γ*_ = *T*_6,*γ*_ = *T*_7,*γ*_ = 0 15:   Call Algorithm 116:   Calculate the jerk of each phase of the S-curve using Equation (11)17:  **otherwise** *v_s,γ_* > *v_e,γ_*18:   The acceleration block does not exist19:   Set *T*_1,*γ*_ = *T*_2,*γ*_ = *T*_3,*γ*_ = 020:   Call Algorithm 121:   Calculate the jerk of each phase of the S-curve using Equation (13)22:  **otherwise** *v_s,γ_* = *v_e,γ_*23:   Acceleration and deceleration blocks do not exist24:   Set *T*_1,*γ*_ = *T*_2,*γ*_ = *T*_3,*γ*_ = *T*_5,*γ*_ = *T*_6,*γ*_ = *T*_7,*γ*_ = 0 and *T*_4,*γ*_ = *L_γ_*/*v_s,γ_*25:   Set the jerk of each phase of the S-curve as zero26:
  **end if**
27:
 **end if**
28: Calculate the acceleration of each phase using Equation (3)29: Calculate the velocity of the *γ*th NURBS segment by integrating acceleration30: Determine the interpolation parameter *u* using Equation (28)31: Determine the NURBS path using Equations (26) and (27)32:**end for**33:Derive the asymmetric S-curve trajectory

## 4. Simulation

Simulations were performed to confirm the feasibility and effectiveness of the proposed time-optimal asymmetric S-curve (TOAS) trajectory planning approach. A comparison with BLA [35] and TSS Acc/Dec control algorithm [37] also demonstrated the efficiency of the proposed method. Moreover, the trajectories generated by the end-effector trial motion profile (TMP) method are analyzed to illustrate the effect of kinematic constraint handling.

**Figure 3:**
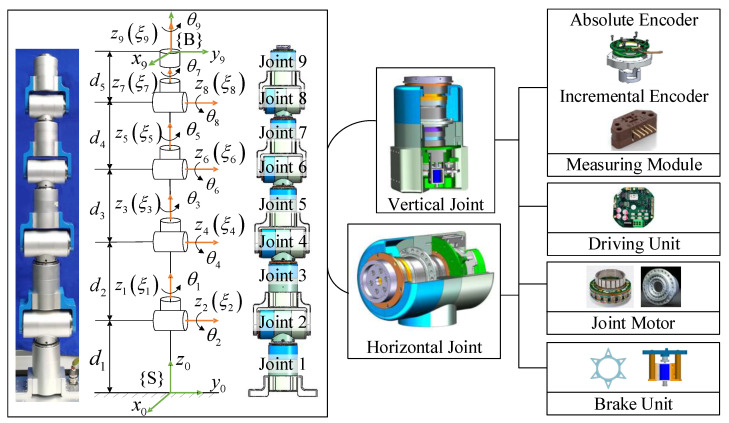
Mechanical structure and kinematic model of the 9-DOF manipulator. {S} is the base frame and {B} is the frame fixed on the end−effector.

A 9-DOF redundant manipulator, self-developed in our laboratory, was employed for simulation. Figure 3 shows the mechanical structure and kinematic model. The representation of forward kinematics depends on the product of the exponential equations, and the related parameters are recorded in Table 1.

Moreover, a virtual model of the robot is used in the simulation without considering the actual control loop performance. For better illustration, the limits of each joint were set to:

(40) $$\begin{array}{c} q_{\lim}=\left(\pi ,\pi /2,\pi ,\pi /2,\pi ,\pi /2,\pi ,\pi /2,\pi \right)\;\mathrm{rad}\\ {\dot{q}}_{\lim}=\left(0.8,0.8,0.9,0.9,0.9,0.9,1,1,1\right)\;rad/s\\ {\ddot{q}}_{\lim}=\left(2,2,3,3,3,3,5,5,5\right)\;rad/s^{2} \end{array}.$$

**Figure 4:**
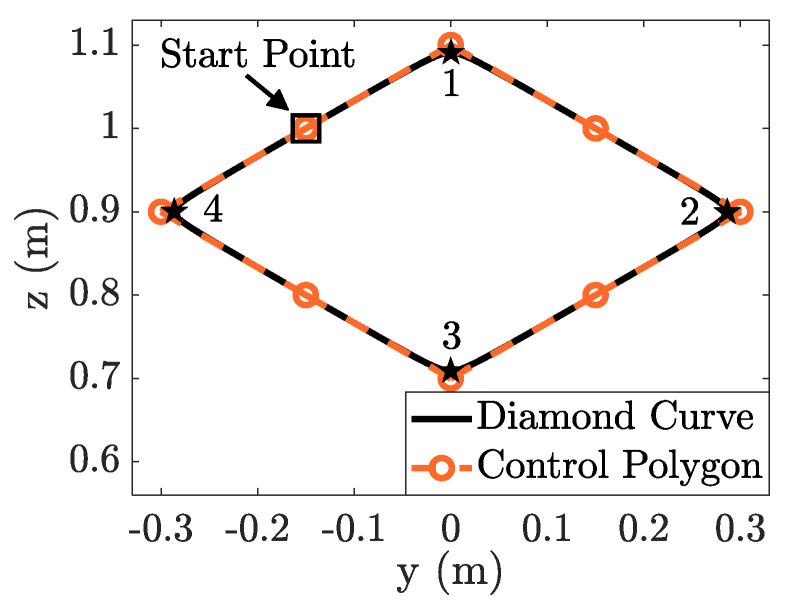
Diamond-shaped curve constructed from the NURBS curve.

The end-effector path employed a diamond-shaped curve constructed from the NURBS curve, as shown in Figure 4. The orientation of the end-effector holds along the entire path. The knot vector, control points, and weight of each control point are listed in Table A1 in Appendix B.

**Figure 5:**
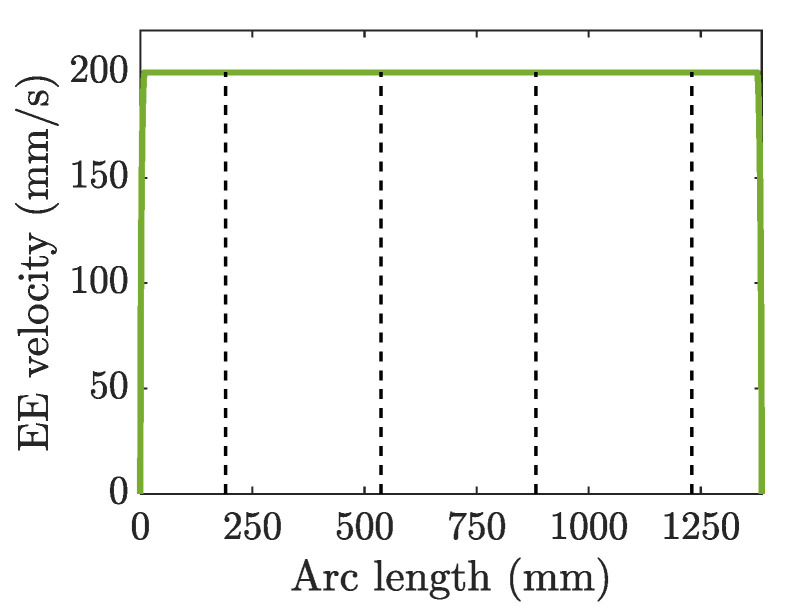
End-effector (EE) trial motion profile along the diamond-shaped NURBS path. The dashed black lines divide the arc length at the four corners of the diamond-shaped NURBS path.

**Figure 6:**
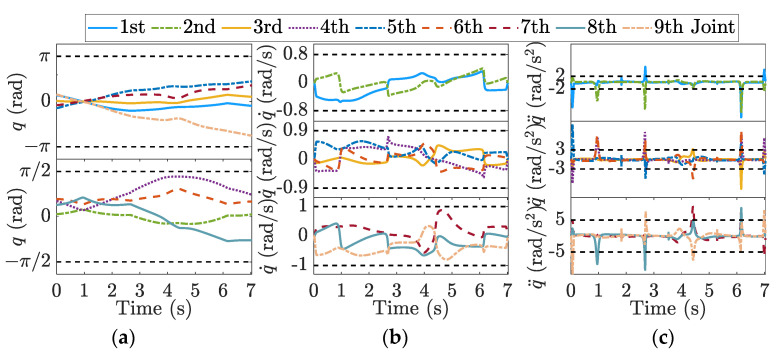
Nine-DOF joint trajectories of the trial motion profile: (**a**) joint positions; (**b**) joint velocities; (**c**) joint accelerations. Dotted grey line: the limits of joint position, velocity, and acceleration.

The closed-loop inverse kinematics algorithm is used to achieve kinematic mapping from end-effector to joints; more details can be found in [40]. When the end-effector trial motion profile is designed based on constraints in operational space, as shown in Figure 5, the joint trajectories in Figure 6 can be derived.

The joint positions are bounded within limits owing to redundancy. Although most of the joint velocities are less than the maximum allowed value, joint acceleration overruns occur in the start–stop phase and the four corners of the diamond curve with a large centripetal acceleration, as shown in Figure 6c. Hence, velocity scheduling based only on the end-effector limitations is infeasible for a robot with complicated kinematics. Robotic capabilities are underutilized in some potential places, whereas the possibility of joint constraint violations exists in dangerous places.

**Figure 7:**
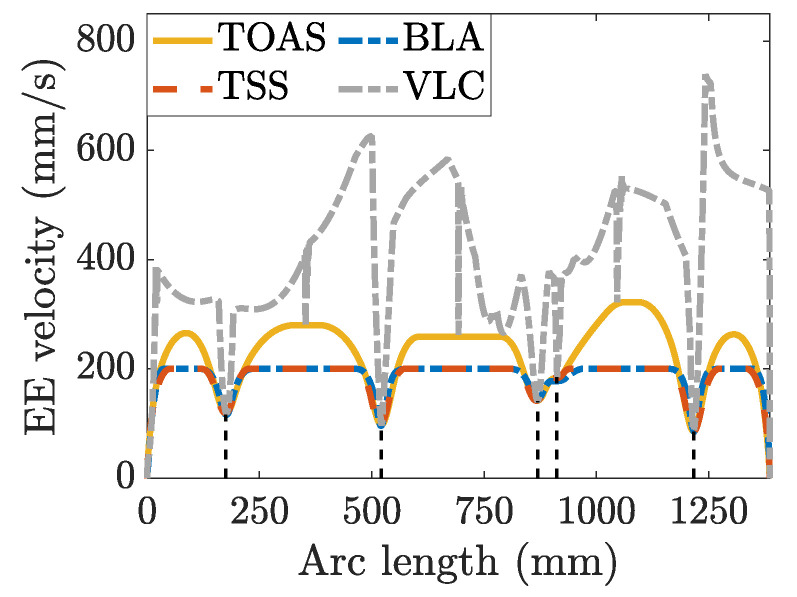
End-effector (EE) velocity profile for different velocity scheduling methods: TOAS (solid yellow line); BLA (dotted blue line); TSS (dashed red line). The dotted grey line is the VLC along the diamond path. The dashed black lines divide the NURBS path into six segments according to the VLC.

In this study, the joint motion shown in Figure 6 is a priori for deriving the end-effector velocity-limit curve. The diamond curve was divided into six segments at the local minimum of the velocity-limit curve. Velocity scheduling was performed for each segment, and the results are shown in Figure 7.

The proposed TOAS method can flexibly deal with the complex constraints of redundant robots, while BLA and TSS require bidirectional scanning from the local minima velocity to satisfy the constraints. Moreover, TSS derives the velocity profile of each segment from the end-effector velocity, acceleration, and jerk limits in the operation space, whereas the TOAS and BLA are guided by the kinematic constraints of the joint actuators. Because the end-effector limitation is related to the configuration of the serial manipulator, as joint motors drive it, a motion profile based on joint mobility can be designed. However, BLA emphasizes velocity scale-down when joint constraints are violated. In contrast, TOAS allows increasing the end-effector velocity when the joint velocity and acceleration have not reached limits. Moreover, TOAS also considers path constraints. The end-effector velocity of the TOAS may not achieve the maximum tolerable velocity if the arc length is not long enough, such as in the motion profiles of the first and sixth segments in Figure 7.

**Figure 8:**
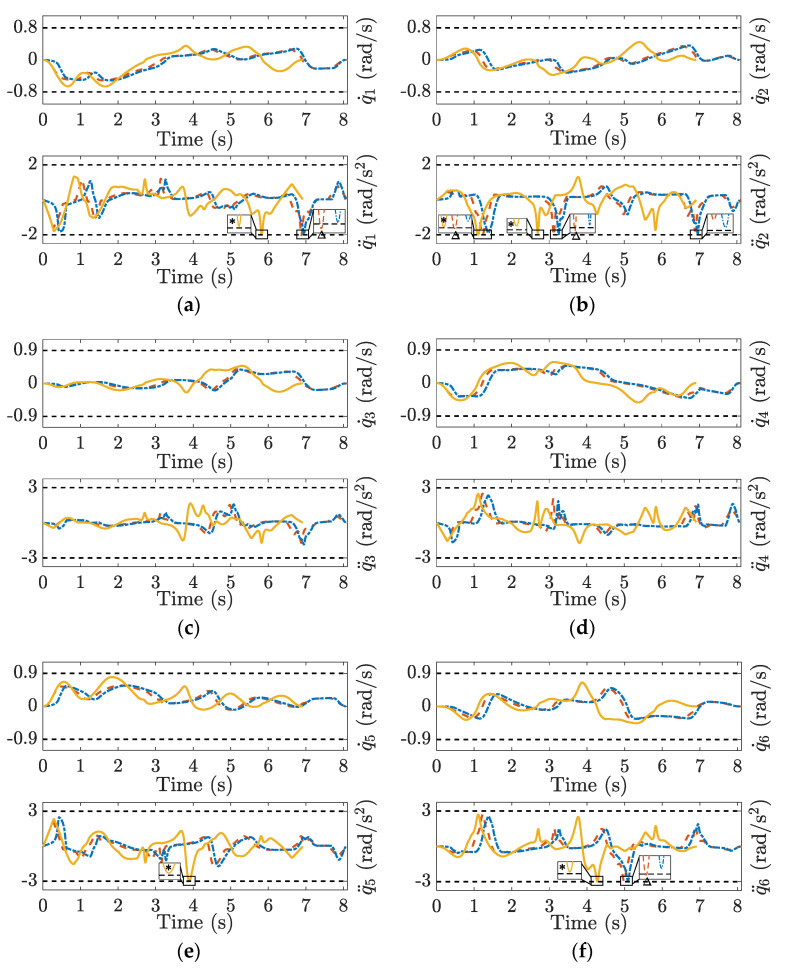

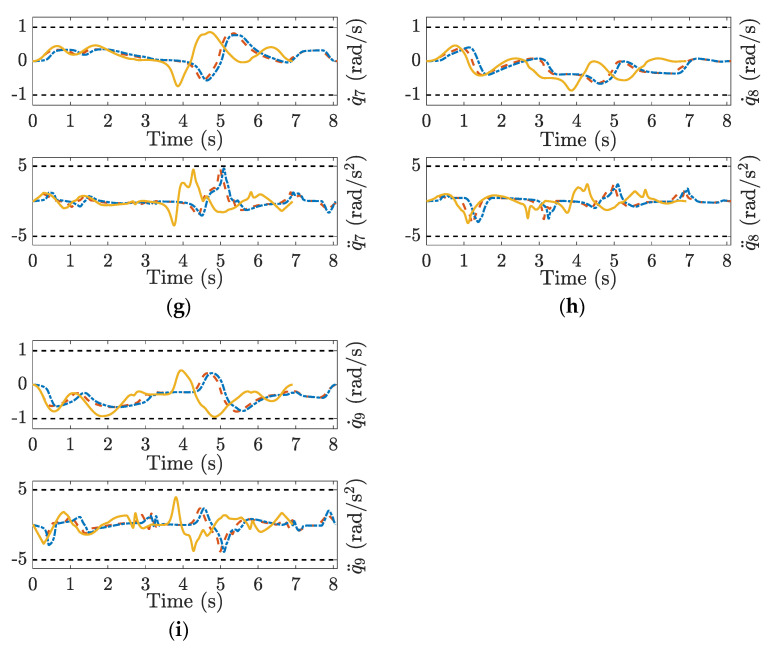
Nine-DOF joint velocity and acceleration profile for different velocity scheduling methods, and (**a**–**i**) correspond to joints 1–9. Solid yellow line: TOAS; dotted blue line: BLA; dashed red line: TSS; dotted grey line: the limits of the joint velocity and acceleration. * represents the motion curves of TOAS nearing limits. Δ represents the motion curves of TSS violating limits.

**Figure 9:**
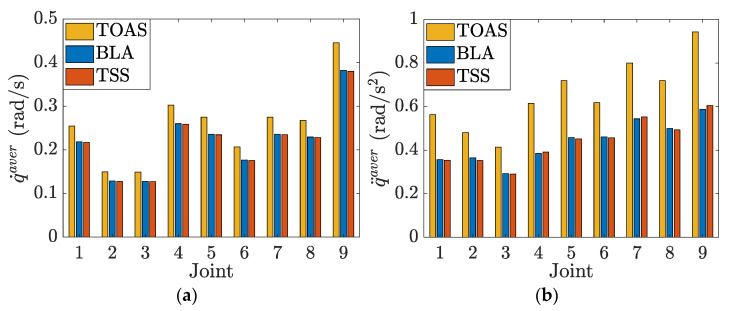
Average values of joint velocity and acceleration for the different velocity scheduling methods. (**a**) Average velocity of each joint; (**b**) average acceleration of each joint.

The joint velocity and acceleration curves for the three methods are plotted in Figure 8. The TOAS and BLA consider the joint velocity and acceleration limits during velocity planning. The movements of all joints were confined to the permissible range. TSS provides a similar end-effector motion profile but causes joint constraint violation because only limits in the operation space are considered. Joints 1, 2, and 6 exhibit different degrees of joint acceleration overrun, as observed from the dashed red line in the partial enlargements of Figure 8a,b,f, respectively. Owing to the prior, the velocity scheduling for each segment starts from the local minimum of the end-effector velocity-limit curve. Consequently, the TSS has no serious limit exceedance as TMP, which also only pays attention to the bounds imposed on the end-effector. In addition, TOAS can provide more efficient planning results than BLA and TSS. For a visual comparison, bar charts of the average joint velocity and acceleration are displayed in Figure 9.

TOAS adopts a metaheuristic algorithm to search for time-optimal kinematic parameters within the acceleration and velocity limits of joint actuators to reduce the execution time along the path. As shown in Table 2, when enforcing the same geometric path, BLA and TSS require similar amounts of time, whereas TOAS takes less time and even less time than the TMP situation.

Table 3 and Table 4 enumerate the position and orientation errors of the end-effector. The position error indicates the Euclidean metric between the designed and measured positions of the end effector, which is described in Cartesian coordinates. The orientation error represents the difference in the Euler angles between the measured attitude and the specified one, which must be perpendicular to the yz-plane. TMP suffers from large trajectory-following errors as the kinematic constraints are violated. TSS can maintain a high tracking performance overall but causes large errors when the joint accelerations are slightly out of bounds. TOAS exhibits similar precision to BLA and TSS while providing faster planning results.

## 5. Experimental Results

Experiments were carried out on the nine-DOF redundant manipulator to illustrate the practicability of the proposed TOAS trajectory planning approach. As shown in Figure 3, the robot features two types of vertical and horizontal joints with nine revolute joints. Each joint consists of a measuring module, driving unit, joint motor, and brake unit. The joint motor is controlled by the driving unit, which communicates with the PC-based control system using the EtherCAT protocol. The control system delivers commands to the robot via TwinCAT automation software at a sampling rate of 200 HZ. Joint information can be collected by a measuring module including two built-in encoders, with a 1000 Hz measurement frequency, which is higher than the control-signal transmission frequency. The absolute encoder collects position information, whereas the incremental encoder returns changes in position, indicating joint velocities. The acceleration values were calculated using numerical differentiation.

The motion system was restricted by the robotic mechanical structure and bandwidth of the control loop. The bandwidth of the velocity loop was 18 Hz. For security purposes, the velocity and acceleration limitations of each joint are set to 0.1 rad/s and 0.5 rad/s^2^, respectively.

**Figure 10:**
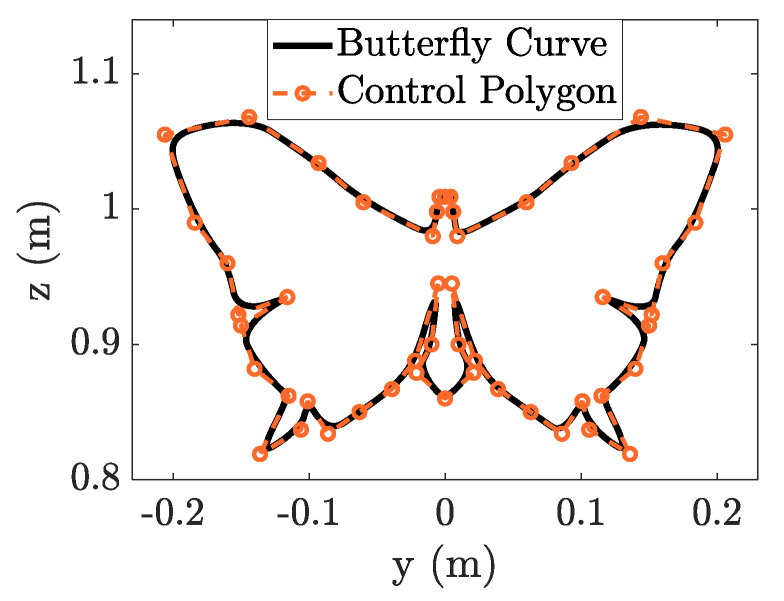
Butterfly-shaped curve constructed from the NURBS curve.

The end-effector path is defined as a butterfly-shaped NURBS curve, as shown in Figure 10. The relevant parameters are listed in Table A2 of Appendix B. The total length of the geometric path is 1.62 m. Specifying the tangential velocity of the end effector as 15 mm/s; the TMP trajectory required 108.885 s to execute. The trial joint trajectories can be obtained as the prior using the closed-loop inverse kinematics algorithm.

**Figure 11:**
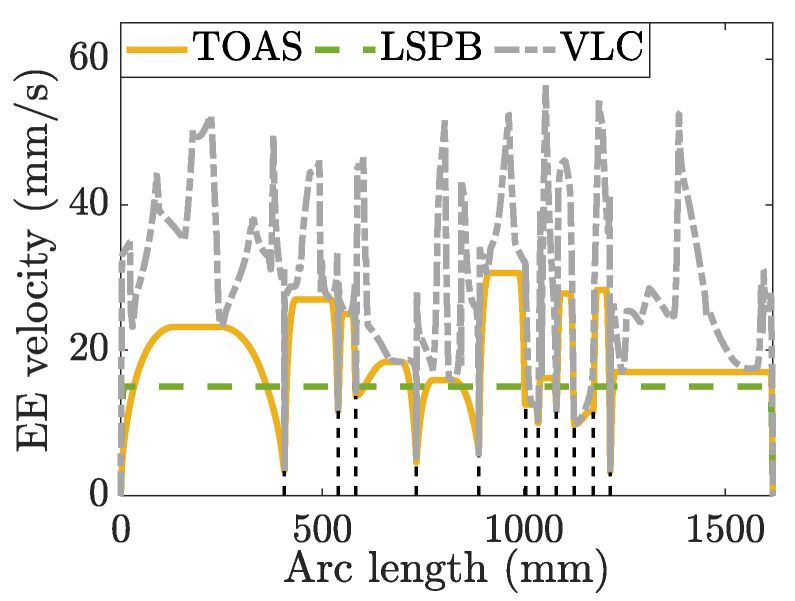
End-effector (EE) motion profile based on the time-optimal S-curve trajectory planning method. The dotted gray line is the VLC along the butterfly path. The dashed black lines divide the NURBS path into thirteen segments according to the VLC.

The path was divided into thirteen segments based on the velocity limits along the butterfly-shaped NURBS curve. The end-effector velocity of each segment was designed using the WOA-based S-curve velocity-scheduling algorithm, and the motion profile along the path is depicted in Figure 11. The design velocity decreases if the joint constraint is violated at a specified constant velocity. If possible, an increase in the design velocity is allowed under the updated velocity limit, which is the maximum allowed end-effector velocity along the path within the joint motion constraints.

**Figure 12:**
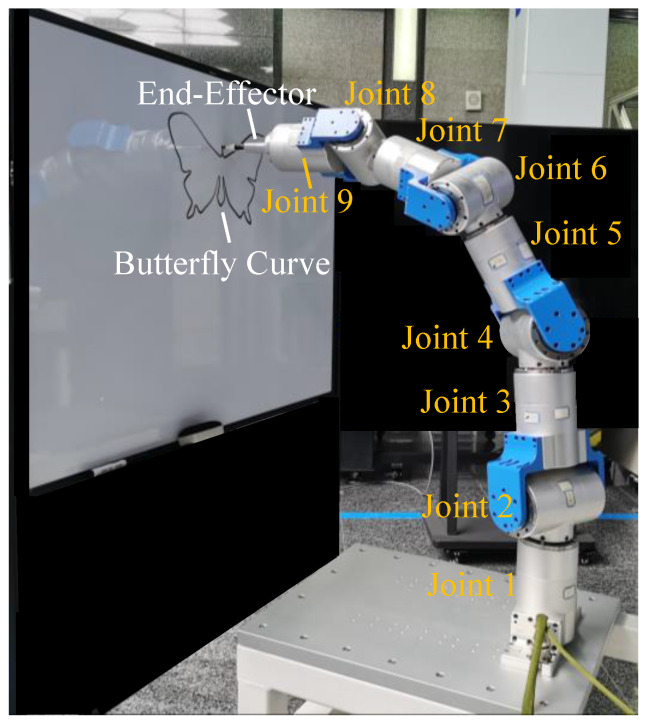
Experimental results.

The designed trajectory of the end effector is located on the yz-plane, 0.7 m along the positive x-axis of the base frame {S}, where the whiteboard should be placed. Figure 12 shows the results of the trajectory-tracking experiment.

**Figure 13:**
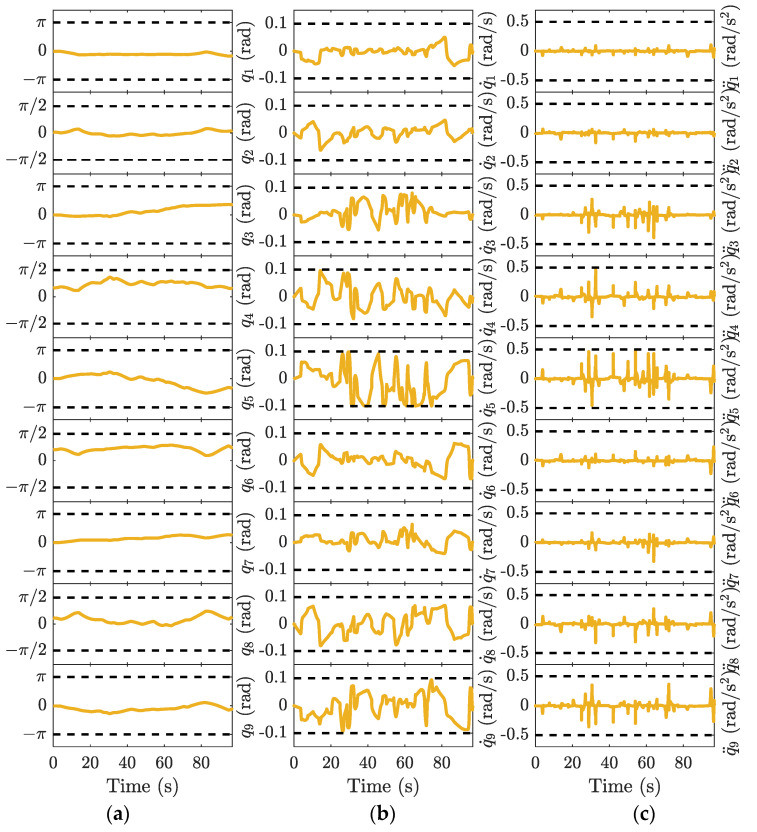
Nine-DOF joint motion profiles for the proposed time-optimal asymmetric S-curve trajectory planning method. Dotted grey line: the limits of the joint position, velocity, and acceleration in (**a**) joint position; (**b**) joint velocity; and (**c**) joint acceleration.

The position, velocity, and acceleration profiles of each joint derived using the inverse kinematics are shown in Figure 13. The proposed method provides a reasonable joint motion within the allowable range. In addition, TOAS takes 96.755 s to complete the butterfly curve, while TSS and BLA require 113.055 s and 113.195 s, respectively. However, the motion accuracy of the experiment is worse than that simulated on an ideal model because of the influence of the actual controller and intrinsic mechanical structure. The trajectory tracking errors, including the mean position and orientation errors, are listed in Table 5. The TMP causes large path deformations, where the planning result violates the kinematic constraints, saturating the joint motors. The tracking error of the TOAS is of the same magnitude as that of the BLA and TSS. The results illustrate that TOAS provides faster motion than the other two methods while guaranteeing high trajectory tracking performance.

## 6. Conclusions

We have introduced a TOAS trajectory planning approach to achieve the fast and smooth motion requirements of a specified end-effector path within the kinematic constraints. According to a velocity-limit curve along the specified path, asymmetric S-curve velocity scheduling is performed in segments. The WOA finds the optimal S-shape motion profile. Moreover, it requires no assumption to generate a TOAS for paths with arbitrary arc length and start-end velocity. The metaheuristic WOA improves the flexibility of constraint processing and avoids deciding the deceleration block onset. The feasibility and effectiveness of the method were verified through suitable simulations and experiments on a redundant manipulator. The task execution time along the same NURBS path of the TOAS was approximately 14.5% less than that with BLA and TSS. The time-optimal motion profile with high-accuracy trajectory tracking is designed based on the kinematic constraints of the joint actuators, unlocking the robotic capabilities. The proposed approach can be applied to serial manipulators adopting a numerical method to solve inverse kinematic problems, generating smooth trajectories, and reducing task execution time. However, the TOAS method is offline-based and requires a set of trial joint trajectories as priors to impose joint constraints on the end-effector. In the future, we will explore a real-time approach to address joint velocity and acceleration bounds when designing trajectories in the operation space.

## Figures and Tables

**Table 1:** Kinematics parameters of the 9-DOF manipulator.

i	Screw Axis	Configuration at Zero Position	Parameter Values (m)
1	$\xi_{1}=\left[\begin{array}{cccccc} 0; & 0; & 1; & 0; & 0; & 0; \end{array}\right]$	$M_{0}=\left[\begin{array}{cc} \begin{array}{cc} 1 & 0\\ 0 & 1 \end{array} & \begin{array}{cc} 0 & 0\\ 0 & 0 \end{array}\\ \begin{array}{cc} 0 & 0\\ 0 & 0 \end{array} & \begin{array}{cc} 1 & d\\ 0 & 1 \end{array} \end{array}\right]$	$\begin{array}{l} d_{1}=0.2878\\ d_{2}=0.3498\\ d_{3}=0.3187\\ d_{4}=0.2940\\ d_{5}=0.2350\\ d=d_{1}+d_{2}+d_{3}+d_{4}+d_{5} \end{array}$
2	$\xi_{2}=\left[\begin{array}{cccccc} -1; & 0; & 0; & -d_{1}; & 0; & 0; \end{array}\right]$		
3	$\xi_{3}=\left[\begin{array}{cccccc} 0; & 0; & 1; & 0; & 0; & 0; \end{array}\right]$		
4	$\xi_{4}=\left[\begin{array}{ccccccc} -1; & 0; & 0; & -d_{1} & -d_{2}; & 0; & 0; \end{array}\right]$		
5	$\xi_{5}=\left[\begin{array}{cccccc} 0; & 0; & 1; & 0; & 0; & 0; \end{array}\right]$		
6	$\xi_{6}=\left[\begin{array}{cccccccc} -1; & 0; & 0; & -d_{1} & -d_{2} & -d_{3}; & 0; & 0; \end{array}\right]$		
7	$\xi_{7}=\left[\begin{array}{cccccc} 0; & 0; & 1; & 0; & 0; & 0; \end{array}\right]$		
8	$\xi_{8}=\left[\begin{array}{ccccccccc} -1; & 0; & 0; & -d_{1} & -d_{2} & -d_{3} & -d_{4}; & 0; & 0; \end{array}\right]$		
9	$\xi_{9}=\left[\begin{array}{cccccc} 0; & 0; & 1; & 0; & 0; & 0; \end{array}\right]$		

**Table 2:** Execution time for different velocity scheduling methods.

Method	TMP	TOAS	BLA	TSS
Time (s)	7.030	6.910	8.055	8.100

**Table 3:** Position errors for different velocity scheduling methods.

Method	TMP	TOAS	BLA	TSS
Maximum position errors (m)	7.67 × 10^−3^	3.53 × 10^−4^	2.29 × 10^−4^	7.08 × 10^−4^
Mean position errors (m)	4.25 × 10^−4^	5.37 × 10^−5^	4.44 × 10^−5^	6.23 × 10^−5^

**Table 4:** Orientation errors for different velocity scheduling methods.

Method	TMP	TOAS	BLA	TSS
Maximum orientation errors (rad)	9.34 × 10^−3^	7.93 × 10^−5^	7.06 × 10^−5^	1.17 × 10^−4^
Mean orientation errors (rad)	6.69 × 10^−4^	2.95 × 10^−6^	1.15 × 10^−6^	8.44 × 10^−6^

**Table 5:** Trajectory tracking errors for different velocity scheduling methods.

Method	TMP	TOAS	BLA	TSS
Mean position errors (m)	2.82 × 10^−2^	7.92 × 10^−4^	6.38 × 10^−4^	1.21 × 10^−3^
Mean orientation errors (rad)	8.13 × 10^−3^	3.24 × 10^−4^	1.75 × 10^−4^	6.67 × 10^−4^

## Data Availability

The data that support the findings of this study are available from the corresponding author upon reasonable request.

## Appendix A

The velocity–time equation *v*(*t*) is derived by integrating acceleration, which can be written in the following form:

(A1) $$v\left(t\right)=\{\begin{array}{ll} \frac{1}{2}J_{1}t^{2}+v_{s} & 0\leq t\leq \tau_{1}\\ J_{1}\tau_{1}t-\frac{1}{2}J_{1}\tau_{1}^{2}+v_{s} & \tau_{1}\leq t\leq \tau_{2}\\ -\frac{1}{2}J_{3}t^{2}+(J_{3}\tau_{2}+J_{1}\tau_{1})t-\frac{1}{2}J_{3}\tau_{2}^{2}-\frac{1}{2}J_{1}\tau_{1}^{2}+v_{s} & \tau_{2}\leq t\leq \tau_{3}\\ -\frac{1}{2}J_{3}(\tau_{3}^{2}+\tau_{2}^{2})-\frac{1}{2}J_{1}\tau_{1}^{2}+(J_{3}\tau_{2}+J_{1}\tau_{1})\tau_{3}+v_{s} & \tau_{3}\leq t\leq \tau_{4}\\ \begin{array}{l} -\frac{1}{2}J_{5}t^{2}+J_{5}\tau_{4}t-\frac{1}{2}J_{5}\tau_{4}^{2}-\frac{1}{2}J_{3}(\tau_{3}^{2}+\tau_{2}^{2})\\ -\frac{1}{2}J_{1}\tau_{1}^{2}+(J_{3}\tau_{2}+J_{1}\tau_{1})\tau_{3}+v_{s} \end{array} & \tau_{4}\leq t\leq \tau_{5}\\ \begin{array}{l} \left(-J_{5}\tau_{5}+J_{5}\tau_{4})t+\frac{1}{2}J_{5}(\tau_{5}^{2}-\tau_{4}^{2}\right)\\ -\frac{1}{2}J_{3}(\tau_{3}^{2}+\tau_{2}^{2})-\frac{1}{2}J_{1}\tau_{1}^{2}+(J_{3}\tau_{2}+J_{1}\tau_{1})\tau_{3}+v_{s} \end{array} & \tau_{5}\leq t\leq \tau_{6}\\ \begin{array}{l} \frac{1}{2}J_{7}t^{2}+(-J_{7}\tau_{6}-J_{5}\tau_{5}+J_{5}\tau_{4})t+\frac{1}{2}J_{7}\tau_{6}^{2}+\frac{1}{2}J_{5}(\tau_{5}^{2}-\tau_{4}^{2})\\ -\frac{1}{2}J_{3}(\tau_{3}^{2}+\tau_{2}^{2})-\frac{1}{2}J_{1}\tau_{1}^{2}+(J_{3}\tau_{2}+J_{1}\tau_{1})\tau_{3}+v_{s} \end{array} & \tau_{6}\leq t\leq \tau_{7} \end{array}$$

The position–time equation *x*(*t*) is deduced from its integral relationships with velocity, which can be written in the following form:

(A2) $$x\left(t\right)=\{\begin{array}{ll} \frac{1}{6}J_{1}t^{3}+v_{s}t & 0\leq t\leq \tau_{1}\\ \frac{1}{2}J_{1}\tau_{1}t^{2}+(-\frac{1}{2}J_{1}\tau_{1}^{2}+v_{s})t+\frac{1}{6}J_{1}\tau_{1}^{3} & \tau_{1}\leq t\leq \tau_{2}\\ \begin{array}{l} -\frac{1}{6}J_{3}t^{3}+\frac{1}{2}(J_{3}\tau_{2}+J_{1}\tau_{1})t^{2}+(-\frac{1}{2}J_{3}\tau_{2}^{2}\\ -\frac{1}{2}J_{1}\tau_{1}^{2}+v_{s})t+\frac{1}{6}J_{3}\tau_{2}^{3}+\frac{1}{6}J_{1}\tau_{1}^{3} \end{array} & \tau_{2}\leq t\leq \tau_{3}\\ \begin{array}{l} (-\frac{1}{2}J_{3}(\tau_{3}^{2}+\tau_{2}^{2})-\frac{1}{2}J_{1}\tau_{1}^{2}+J_{3}\tau_{3}\tau_{2}+J_{1}\tau_{3}\tau_{1}+v_{s})t\\ +\frac{1}{3}J_{3}\tau_{3}^{3}+\frac{1}{6}J_{3}\tau_{2}^{3}+\frac{1}{6}J_{1}\tau_{1}^{3}-\frac{1}{2}J_{3}\tau_{3}^{2}\tau_{2}-\frac{1}{2}J_{1}\tau_{3}^{2}\tau_{1} \end{array} & \tau_{3}\leq t\leq \tau_{4}\\ \begin{array}{l} -\frac{1}{6}J_{5}t^{3}+\frac{1}{2}J_{5}\tau_{4}t^{2}+(-\frac{1}{2}J_{5}\tau_{4}^{2}-\frac{1}{2}J_{3}(\tau_{3}^{2}+\tau_{2}^{2})\\ -\frac{1}{2}J_{1}\tau_{1}^{2}+J_{3}\tau_{3}\tau_{2}+J_{1}\tau_{3}\tau_{1}+v_{s})t+\frac{1}{6}J_{5}\tau_{4}^{3}+\frac{1}{3}J_{3}\tau_{3}^{3}\\ +\frac{1}{6}J_{3}\tau_{2}^{3}+\frac{1}{6}J_{1}\tau_{1}^{3}-\frac{1}{2}J_{3}\tau_{3}^{2}\tau_{2}-\frac{1}{2}J_{1}\tau_{3}^{2}\tau_{1} \end{array} & \tau_{4}\leq t\leq \tau_{5}\\ \begin{array}{l} -\frac{1}{2}J_{5}(\tau_{5}-\tau_{4})t^{2}+(\frac{1}{2}J_{5}(\tau_{5}^{2}-\tau_{4}^{2})-\frac{1}{2}J_{3}(\tau_{3}^{2}+\tau_{2}^{2})\\ -\frac{1}{2}J_{1}\tau_{1}^{2}+J_{3}\tau_{3}\tau_{2}+J_{1}\tau_{3}\tau_{1}+v_{s})t+\frac{1}{6}J_{5}(-\tau_{5}^{3}+\tau_{4}^{3})\\ +\frac{1}{3}J_{3}\tau_{3}^{3}+\frac{1}{6}J_{3}\tau_{2}^{3}+\frac{1}{6}J_{1}\tau_{1}^{3}-\frac{1}{2}J_{3}\tau_{3}^{2}\tau_{2}-\frac{1}{2}J_{1}\tau_{3}^{2}\tau_{1} \end{array} & \tau_{5}\leq t\leq \tau_{6}\\ \begin{array}{l} \frac{1}{6}J_{7}t^{3}+\frac{1}{2}(-J_{7}\tau_{6}-J_{5}\tau_{5}+J_{5}\tau_{4})t^{2}+(\frac{1}{2}J_{7}\tau_{6}^{2}\\ +\frac{1}{2}J_{5}(\tau_{5}^{2}-\tau_{4}^{2})-\frac{1}{2}J_{3}(\tau_{3}^{2}+\tau_{2}^{2})-\frac{1}{2}J_{1}\tau_{1}^{2}+J_{3}\tau_{3}\tau_{2}\\ +J_{1}\tau_{3}\tau_{1}+v_{s})t-\frac{1}{6}J_{7}\tau_{6}^{3}+\frac{1}{6}J_{5}(-\tau_{5}^{3}+\tau_{4}^{3})+\frac{1}{3}J_{3}\tau_{3}^{3}\\ +\frac{1}{6}J_{3}\tau_{2}^{3}+\frac{1}{6}J_{1}\tau_{1}^{3}-\frac{1}{2}J_{3}\tau_{3}^{2}\tau_{2}-\frac{1}{2}J_{1}\tau_{3}^{2}\tau_{1} \end{array} & \tau_{6}\leq t\leq \tau_{7} \end{array}$$

## Appendix B

Table A1 lists the parameters of the diamond-shaped NURBS curve in Figure 4, including the degree, knot vector, control points, and weight of each control point.

**Table A1:** Parameters of t diamond-shaped curve.

Items	Parameters
Degree: *p*	2
Knot vector: *U*_1×12_	0, 0, 0, 0.25, 0.25, 0.50, 0.50, 0.75, 0.75, 1.00, 1.00, 1.00
Weights: *ω*_1×9_	1.00, 10.00, 1.00, 10.00, 1.00, 10.00, 1.00, 10.00, 1.00
Control points: *d*_9×3_(m)	(0.70, −0.15, 1.00); (0.70, 0, 1.10); (0.70, 0.15, 1.00); (0.70, 0.30, 0.90); (0.70, 0.15, 0.80); (0.70, 0, 0.70); (0.70, −0.15, 0.80); (0.70, −0.30, 0.90); (0.70, −0.15, 1.00);

Table A2 lists the parameters of the butterfly-shaped NURBS curve in Figure 10, including the degree, knot vector, control points, and weight of each control point.

**Table A2:** Parameters of a butterfly-shaped curve.

Items	Parameters
Degree: *p*	2
Knot vector: *U*_1×55_	0, 0, 0, 0, 0.0083, 0.0150, 0.0361, 0.0855, 0.1293, 0.1509, 0.1931, 0.2273, 0.2435, 0.2561, 0.2692, 0.2889, 0.3170, 0.3316, 0.3482, 0.3553, 0.3649, 0.3837, 0.4005, 0.4269, 0.4510, 0.4660, 0.4891, 0.5000, 0.5109, 0.5340, 0.5489, 0.5731, 0.5994, 0.6163, 0.6351, 0.6447, 0.6518, 0.6683, 0.6830, 0.7111, 0.7307, 0.7439, 0.7565, 0.7729, 0.8069, 0.8491, 0.8707, 0.9145, 0.9639, 0.9850, 0.9917, 1, 1, 1, 1
Weights: *ω*_1×55_	1.00, 1.00, 1.00, 1.20, 1.00, 1.00, 1.00, 1.00, 1.00, 1.00, 1.00, 2.00, 1.00, 1.00, 5.00, 3.00, 1.00, 1.10, 1.00, 1.00, 1.00, 1.00, 1.00, 1.00, 1.00, 1.00, 1.00, 1.00, 1.00, 1.00, 1.00, 1.00, 1.00, 1.10, 1.00, 3.00, 5.00, 1.00, 1.00, 2.00, 1.00, 1.00, 1.00, 1.00, 1.00, 1.00, 1.00, 1.20, 1.00, 1.00, 1.00
Control points: *d*_55×3_(m)	(0.700, 0, 1.009); (0.700, 0.004, 1.009); (0.700, 0.006, 0.998); (0.700, 0.009, 0.980); (0.700, 0.060, 1.005); (0.700, 0.093, 1.034); (0.700, 0.144, 1.068); (0.700, 0.206, 1.055); (0.700, 0.184, 0.990); (0.700, 0.160, 0.960); (0.700, 0.152, 0.922); (0.700, 0.116, 0.935); (0.700, 0.150, 0.914); (0.700, 0.140, 0.882); (0.700, 0.115, 0.862); (0.700, 0.136, 0.819); (0.700, 0.106, 0.837); (0.700, 0.101, 0.858); (0.700, 0.086, 0.834); (0.700, 0.063, 0.850); (0.700, 0.039, 0.867); (0.700, 0.022, 0.888); (0.700, 0.005, 0.945); (0.700, 0.010, 0.900); (0.700, 0.021, 0.879); (0.700, 0, 0.860); (0.700, −0.021, 0.879); (0.700, −0.010, 0.900); (0.700, −0.005, 0.945); (0.700, −0.022, 0.888); (0.700, −0.039, 0.867); (0.700, −0.063, 0.850);(0.700, −0.086, 0.834); (0.700, −0.101, 0.858); (0.700, −0.106, 0.837); (0.700, −0.136, 0.819); (0.700, −0.115, 0.862); (0.700, −0.140, 0.882); (0.700, −0.150, 0.914); (0.700, −0.116, 0.935); (0.700, −0.152, 0.922); (0.700, −0.160, 0.960); (0.700, −0.184, 0.990); (0.700, −0.206, 1.055); (0.700, −0.144, 1.068); (0.700, −0.093, 1.034); (0.700, −0.060, 1.005); (0.700, −0.009, 0.980); (0.700, −0.006, 0.998); (0.700, −0.004, 1.009); (0.700, 0, 1.009);
